# Influence of bionic footwear on lower limb biomechanics across running experience levels: a controlled laboratory study

**DOI:** 10.3389/fspor.2025.1536629

**Published:** 2025-07-31

**Authors:** Xue Li, Enze Shao, Yufei Fang, Dongxu Wang, Zhiyi Zheng, Hairong Chen, Qian Liu, Gusztáv Fekete, Dong Sun, Yaodong Gu

**Affiliations:** ^1^ Department of Rehabilitation, Ningbo No. 2 Hospital, Ningbo, China; ^2^ Faculty of Sports Science, Ningbo University, Ningbo, China; ^3^ Doctoral School on Safety and Security Sciences, Óbuda University, Budapest, Hungary; ^4^ ANTA Sports Science Laboratory, Department of Science and Innovation, ANTA (China) Co., Ltd, Xiamen, China; ^5^ Faculty of Engineering, University of Szeged, Szeged, Hungary; ^6^ Department of Material Science and Technology, Audi Hungarian Faculty of Vehicle Engineering, Széchenyi István University, Győr, Hungary

**Keywords:** footwear, running experience, bionic technique, biomechanics, sport injury

## Abstract

**Introduction:**

While the biomechanics of lower extremity during running and the impact of conventional running shoes on these traits have been extensively investigated, the influence of bionic shoes on runners remains largely, especially those runners with different experience levels. The aim of this study was to evaluate the biomechanical differences between experienced and novice runners when wearing two distinct types of footwear: bionic shoes and neutral shoes.

**Methods:**

Fourteen healthy male heel-strike runners participated and completed the running test wearing two pairs of running shoes respectively. A two-way-repeated-measures analysis of variance was used to determine the effects of participant experience level and shoe type on joint biomechanics. During the stance phase, shoe design primarily influenced the kinematic and dynamic performance of the ankles, knees, and hip joints.

**Results:**

When participants wore bionic shoes, there was a significant increase in the range of motion of the ankle and hip joints (*p* < 0.010), a remarkable increase in knee joint angular velocity (*p* < 0.010), and a significant decrease in hip joint angular velocity (*p* < 0.001). Concerning differences in experience levels, experienced runners exhibited significantly higher ankle joint angular velocity (*p* = 0.005) and knee joint angular velocity (*p* < 0.010) compared to novice runners, whereas novice runners demonstrated a significantly greater range of knee joint motion than experienced runners (*p* < 0.050).

**Conclusion:**

Our findings preliminarily suggest that experienced runners demonstrate superior performance as well as better stability and motor control of knee joint compared to novice runners who showed smaller knee angular velocity and greater range of motion during running. Furthermore, the increased range of motion of the ankle and hip joints in bionic shoes can activate the relevant muscle groups to a greater extent, which have a certain potential effect on the training performance of runners and the improvement of muscle control ability. While, due to the lack of a certain movement foundation, novice runners may have higher risk of injury.

## 1 Introduction

Running, as a popular form of physical activity, has seen a rapid surge in global participation owing to its accessibility and minimal need for equipment (1). Moreover, it is widely recognized for its positive impact on health, including reducing obesity rates, promoting mental health (2, 3), alleviating cardiovascular conditions, and mitigating the risk of various chronic metabolic diseases (4–7). Despite its popularity as an effective way to keep fit, running also carries a certain risk of injury (8–10), especially lower limb (11). Studies showing that up to 79% of runners experience musculoskeletal injuries each year (12, 13), and a large proportion of these are the result of overuse (14). Giving that individuals inevitably make contact with ground and generate counter-acting forces during movement, providing them with preventative shoes (improving the mid-sole support structure of the shoe may be a protective measure) is an effective approach (15, 16).

As the direct interface between the foot and the ground during running, running shoes are uniquely designed to provide additional support, cushioning, and stability. Hence, there is a substantial body of investigation showing that different types of shoes can significantly influence the biomechanical characteristics of runners (17–21). For instance, the midsole of running shoes has a great impact on running performance and intends to cause running-related physical injuries (22, 23). Although there have been studies on the impact of bionic shoes (simulate the barefoot running conditions as much as possible) on the lower limb biomechanics of runners, the unique differences of bionic shoes on runners with different levels are still blank. This study studies the lower limb characteristics of different experienced runners based on bionic shoes with different footwear. Therefore, selecting running shoes that match your foot type, running habits and experience is essential to enhancing performance and reducing the risk of injury (24). Basically, bionic shoes add unstable design elements and scientific design treatment (25), leading the creation of an unstable environment by providing uneven surface, thus it will improve neuromuscular control. Multiple systems such as nervous system and motor system participate in and interact with each other simultaneously, constantly stimulating athletes' central nervous system and improving athletes' stress ability. The concept of unstable shoes is developed on unstable training equipment: the Masai Barefoot Technology (MBT), which consists of a curved sole in antero-posterior direction and a flexible heel (26). The shoes with unstable structural have been becoming popular with the reason of being not only a therapeutic (27), but a functional aid recently (28). And through the study of running shoes and the anatomical structure of human feet, the midsole of running shoes is the core part of running shoes due to its unique cushioning and support functions, which not only affects comfort, but also improves athletic performance. It can effectively cushion the impact of the foot on the landing, while providing enough support to provide necessary protection for the knee and lower limb joints of the foot. The difference between bionic shoes involved in this study and normal shoes lies in the change of midsole structure. There has been a lot of research and discussion about unstable shoes in recent years. Nigg (29) and other colleagues did an experiment of six week's training with MBT shoes, and the result indicated that it didn't improve their balance capacity. Additionally, Zhou et al. (30) investigated the difference of lower limb kinematics and dynamics between normal shoes and bionic shoes during single leg landing task and found that bionic shoes are more inclined to induce bending of knee joint and hip joint compared to neutral shoes in the last 80% stage of the process, thereby potentially diminishing the risk of lower limb injuries. These studies show that the unstable structure of running shoes can change the range of joint movement, increase the degree of muscle participation, thereby improving body control ability and reducing unnecessary injuries in sports activities (25).

On the other hand, running experience is critical factor influencing performance and injury risk among runners as well (31). Different levels of running experience directly affect runners' sports performance and the occurrence of sports injuries. The main reason lies in the nature of running as a prolonged, repetitive activity (32), where continual impacts can produce varying levels of joint damage. Van Mechelen (33) proposed that about 50%–75% of sports injuries may be due to overuse injuries caused by the re-peated repetition of the same action, such as walk, run and bike, etc. Videbæk et al. (34) have demonstrated the incidence of injury per 1,000 h of running, in which the rate of injury was 17.8% of novice runners compared to recreational runners (7.7%) and ultramarathon runners (7.2%). Among all runner populations, novice runner's injury rate was higher compared to recreational, competitive, or marathon runners. The possible reason is that compared with experienced runners, novice runners have less control over their bodies during running, as well as some other external reasons, such as no scientific training, different running environment and ground conditions, and different running habits (7, 35). Some studies have focused on the effects of running shoes on experienced runners (36, 37) to better improve their athletic performance, however, there are relatively few comparative studies on running shoes for runners with different experience levels (38, 39), probably because most novice runners don't pay much attention to equipment, especially the impact of bionic shoes on runners with different experience levels. Therefore, this paper will examine the biomechanics differences in lower limb of novice and experienced runners wearing neutral shoes and bionic shoes, respectively, with focus on kinematic data such as angle and angular velocity, as well as dynamic indicators. By analyzing the biomechanical characteristics of runners at varying experience levels in bionic footwear, this study aims to provide suggestions for selecting personalized footwear for different runners in terms of injury prevention.

## 2 Materials and methods

### 2.1 Participants

The sample size was calculated using G*Power 3.1 (Franz Faul, Germany) for Two-factor analysis of variance for detecting a medium Cohen's effect size of 0.4, *α* error probability = 0.05 and power (1−*β*) = 0.8 (40), and 14 runners were recruited for meeting the simple size. Seven healthy male experienced heel strike runners (Level 1, Mean ± SD: age: 24.40 ± 2.60, height: 1.73 ± 0.02 m, mass: 59.20 ± 1.60 kg, BMI: 20.00 ± 0.40 kg/m^2^, shoe size: 42) and seven healthy male novice heel strike runners (Level 2, mean ± SD: age: 24.60 ± 0.80, height: 1.74 ± 0.04 m, mass: 74.20 ± 4.80 kg, BMI: 24.70 ± 0.80 kg/m^2^, shoe size: 42) were recruited as experimental participants for this study and their dominant leg was the right leg [the dominant leg means the preferred leg when kicking a ball (41)]. Considering the feasibility of dividing novice runners and experienced runners, the two groups of people are defined as follows: the novice runners are defined as individuals without run regularly or formal training (1, 42). About the experienced runners are defined as the person who run a minimum distance of 20 km per week and participated in all kinds of marathon races regularly with personal season best time < 37 min and 30 s in a 10-kilometers running (43). All participants were recruited through social media and the sports clubs of Ningbo University. All participants were free from health issues, neuromuscular disorders, and lower extremity injuries within the past six months. Before the experiment, all the participants were given the informed consent form and signed it. The Ethics Committee of the Ningbo University Research Institute granted approval for this study (RAGH202401193312), which was conducted in adherence to the principles of the Declaration of Helsinki.

### 2.2 Shoes

**Figure 1:**
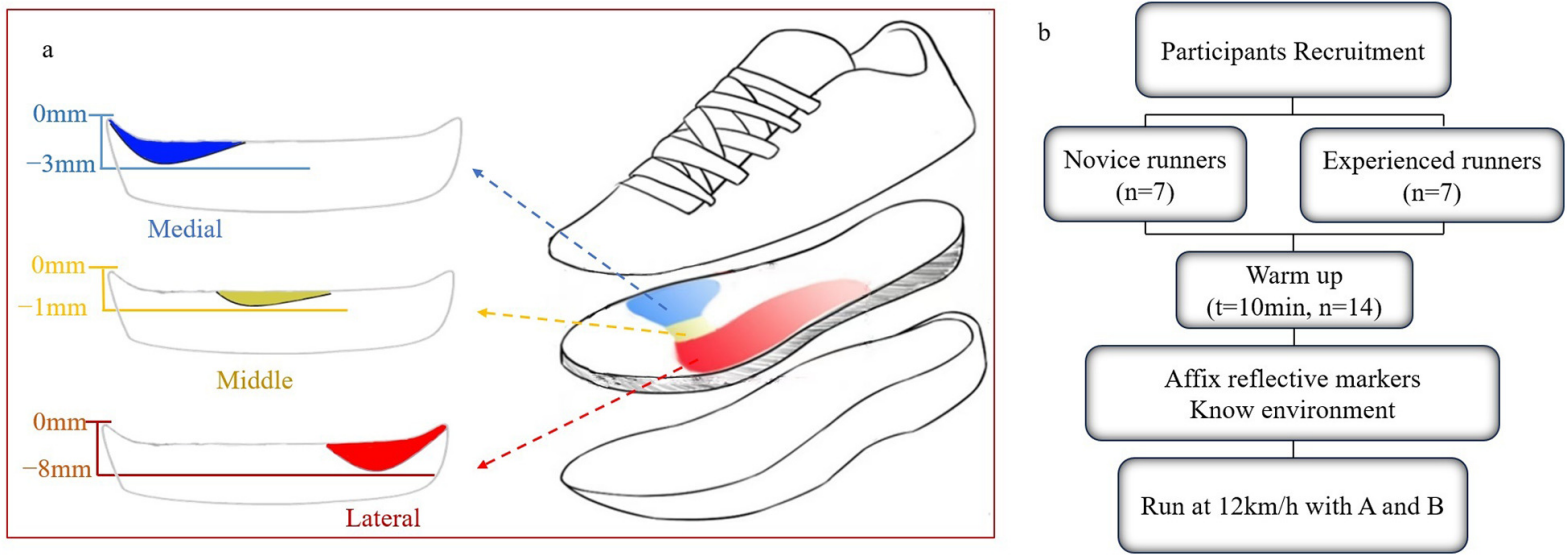
**(a)** Schematic diagram of the stratification of experimental shoes and the illustration of bionic midsole construction; **(b)** flowchart of testing protocol, A means bionic shoes and B means neutral shoes.

Shoes that used in this test include two different kinds: neutral shoes and bionic shoes. Based on neutral shoes design, their primary distinction lies in a thinner midsole to simulate the original state of a person running barefoot more realistically, as illustrated by the colored section in Figure 1a. In this study, the middle part of bionic shoes was removed partly to better simulate the real state of human barefoot running. When a person runs barefoot, the force on the inner and outer sides of the sole is greater, and at the same time, the force on the outer side is the main one. By studying the anatomical structure of feet, a portion of the bionic shoes' midfoot was removed to better simulate human movement characteristics during running. Some other bionic shoes were customized based on individual's foot characteristics, which provided inspiration for our bionic shoes (25). The thicknesses of the removed parts were 3 mm, 1 mm, and 8 mm for blue, yellow, and red separately (as shown in Figure 1a). The main links of the experiment are shown below (Figure 1b).

### 2.3 Experimental procedures

**Figure 2:**
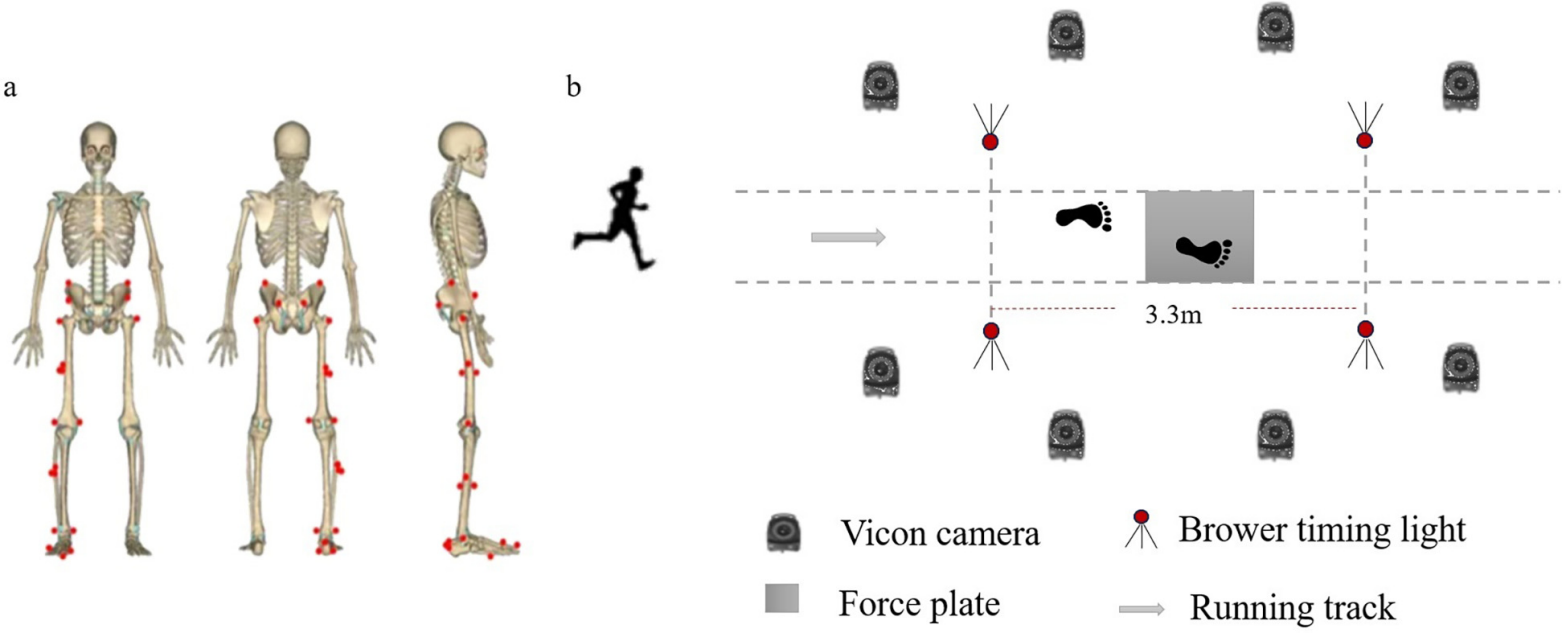
**(a)** Marker placement; **(b)** experimental design for collecting biomechanical data in running stage.

To keep congruity and avoid experimental differences, all participants are required to wear uniform. Before the formal test starts, participants have 10 min to warm-up and familiarize themselves with the process and laboratory apparatus. In preparation, thirty-eight reflective markers were affixed to the body of each participant and rein-forced to ensure they didn't fall and stayed in the same place while running (as shown in Figure 2a). The first step of the experiment was to collect the static coordinates when the participant was standing on the force platform, and then did the running test on the overground runway at the speed of 12.00 ± 0.05 km/h (44, 45). Five successful trials which the dominant leg stepped on the in-ground force plate in the middle of the track were accomplished to gather eligible data (46).

An eight-camera motion capture system (Vicon Metrics Ltd., Oxford, UK) was used to record kinematic data during stance of running at frequency of 200 Hz, and the ground reaction forces was recorded by a force plate (AMTI, Watertown, MA, USA) which was located in the middle of an overground track at 1,000 Hz. Besides, using Brower timing gates (Brower Timing System, Draper, UT, USA) to measure and control the speed of participants on the runway (as shown in Figure 2b). All the tests were conducted in the biomechanics laboratory of Ningbo University.

### 2.4 Data analysis

This study focused on analyzing the kinematic and kinetic characteristics of the hip, knee, and ankle joints and since running is a periodic movement in the forward and backward direction, we mainly study the characteristics of runners' sagittal plane movement in the sagittal plane during running. To accurately capture these variations, marker trajectory data were filtered using a zero-phase fourth-order Butterworth low-pass filter with a cutoff frequency of 12 Hz (47). Subsequently, Matlab R2018a software was utilized to convert the marker trajectory data from C3D file format which were produced by using Vicon Nexus software (version 1.8.5A, Vicon Metrics Ltd., Oxford, UK), to a format compatible with OpenSim 4.3 software (“.mot” and “.trc” formats), and import them into OpenSim for further data processing.

The personal static model was imputed into Opensim 4.3 so that we can obtain the anthropometric model of each participant. The inverse kinematics (IK) and inverse dynamics (ID) tool was used to calculate kinematic and kinetic data. The muscle–skeletal model provided by OpenSim (gait 2392 model) was employed during this process. The inverse kinematics tool was operated to calculate the internal joint angles, and the inverse dynamics tool was operated to calculate the joint moments, and then use excel to calculate the angular velocity and the joint power. The kinetic data were normalized for everyone's body mass. Joint kinematic and kinetic data were time-normalized to 101 datapoints per stand phase using MATLAB version R2018a (The Math Works, Natick, MA, USA) (48).

### 2.5 Statistical analysis

Before statistical analysis, it's essential to do ANOVA assumptions (normality and homogeneity of residuals). If assumptions were met, the two-way repeated-measures was used to evaluate the main effects caused by shoes or running levels, and the inter-actions of the two factors. While if the assumption were not satisfied, a permutation procedure was performed. And alpha level was set at 0.05. When the interaction effect was significant (*p* < 0.050), post-verification comparisons with a Bonferroni correction were carried out *post hoc* to further analyze the significant effects of the running experience, the shoes, and their interaction. Statistical calculations were carried out using SPSS version 27.0 software (SPSS, Chicago, IL, USA). The kinematic and kinetic data during the running stance phase were compared using SPM1D (Statistical Parametric Mapping 1D), a method for analyzing data from one dimensional (1D) spatial domain (49, 50). For SPM1D, descriptive data for each moment were time-normalized to the stance phase (101 data points per stance phase).

## 3 Results

### 3.1 Effects of shoe condition

**Figure 3:**
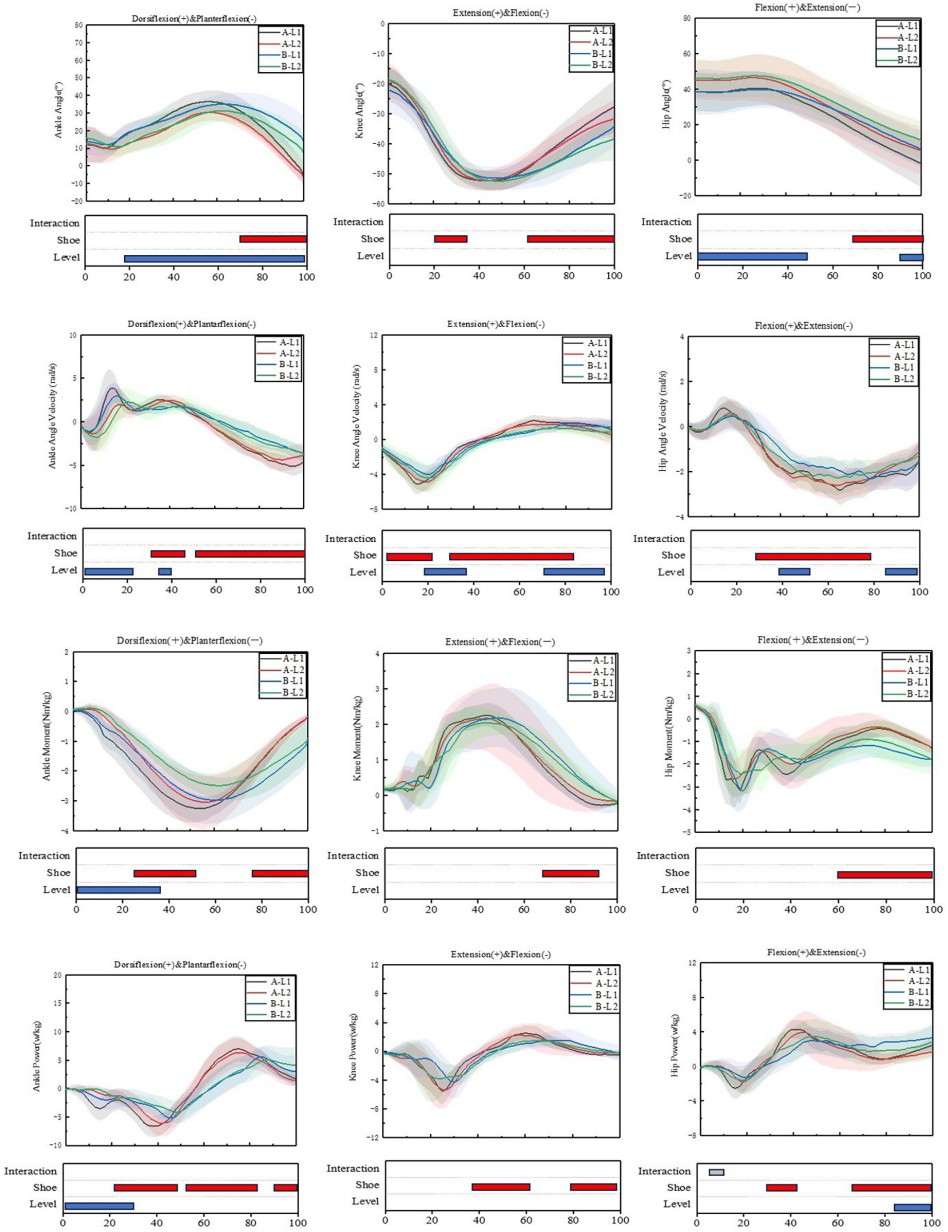
Lower limb joint angle, angle velocity, moment and angle power waveforms of mean and standard deviation over the stance phase for neutral shoes and bionic shoe of two groups of people. A represent Bionic shoes, B represent the natural shoes, and L1 represent the experienced runners, L2 represent the novice runners.

Regarding the shoe condition, we found significant differences between bionic shoes and neutral shoes at the hip, knee, and ankle joints, especially the ankle joint, as shown in Table 1. No matter the novice runners or the experienced runners, the angle range of motion (ROM) of the bionic shoes was greater than neutral shoes in ankle (*F* = 85.651; *p* < 0.010) and hip joint (*F* = 21.311; *p* < 0.010). The changes of angle and angular velocity of ankle, knee and hip joints are shown in Figure 3. The positive work (*F* = 19.815; *p* < 0.010) of ankle also showed significant lower value in neutral shoes. Moreover, the peak angular velocity of the knee joint (*F* = 7.802; *p* < 0.010) showed decreases in neutral shoes at the two groups of people, and the peak angular of the hip joint (*F* = 12.939; *p* < 0.010) showed lower value while wearing bionic shoes.

**Table 1:** Mean (SD)of ROM, peak angular velocity, maximum power, positive work, and negative work of the stance phase during running for the experienced runners and the novice runners with two different shoes.

Joint	Parameters	Group				*p*		
		A/L1	A/L2	B/L1	B/L2	S	L	S*L
Ankle	ROM (°)	42.13 ± 6.04^a,c^	38.17 ± 5.70^b,c^	26.78 ± 3.42^a^	28.09 ± 10.28^b^	***F*** **=** **85.615** ***p*** **<** **0.010**	*F* = 0.932 *p* = 0.337	*F* = 3.673 *p* = 0.058
	Peak angular velocity (rad s^−1^)	11.25 ± 3.55^c^	8.60 ± 3.80^c^	9.99 ± 2.68	8.55 ± 4.03	*F* = 0.839 *p* = 0.362	***F*** **=** **8.222** ***p*** **=** **0.005**	*F* = 0.704 *p* = 0.403
	Peak Moment (N m kg^−1^)	3.18 ± 0.39^c^	2.83 ± 0.73^c^	3.09 ± 0.77^d^	2.57 ± 0.38^d^	*F* = 2.145 *p* = 0.146	***F*** **=** **13.044** ***p*** **<** **0.010**	*F* = 0.498 *p* = 0.482
	Maximum power (W)	18.97 ± 4.34^a^	14.37 ± 4.73^b^	16.35 ± 5.12^a^	13.63 ± 3.62^b^	*F* = 3.466 *p* = 0.066	***F*** **=** **16.465** ***p*** **<** **0.001**	*F* = 1.085 *p* = 0.300
	Positive Work (J kg^−1^)	1.00 ± 0.19^a,c^	0.85 ± 0.29^c^	0.73 ± 0.23^a^	0.73 ± 0.20	***F*** **=** **19.815** ***p*** **<** **0.010**	*F* = 3.055 *p* = 0.084	*F* = 3.005 *p* = 0.084
	Negative Work (J kg^−1^)	0.79 ± 0.17	0.63 ± 0.20	0.68 ± 0.23	0.68 ± 0.23	*F* = 0.372 *p* = 0.543	*F* = 3.787 *p* = 0.055	*F* = 3.787 *p* = 0.055
Knee	ROM (°)	32.98 ± 4.77^c^	33.46 ± 5.56^c^	29.72 ± 6.31	34.35 ± 3.80	*F* = 1.287 *p* = 0.259	***F*** **=** **5.984** ***p*** **=** **0.016**	*F* = 3.930 *p* = 0.050
	Peak angular velocity (rad s−1)	5.15 ± 1.31^c^	4.51 ± 0.54^b,c^	4.93 ± 1.34^d^	3.50 ± 1.01^b,d^	***F*** **=** **7.802** ***p*** **=** **0.006**	***F*** **=** **21.918** ***p*** **<** **0.010**	*F* = 3.323 *p* = 0.071
	Peak Moment (N m kg−1)	2.31 ± 0.31	2.18 ± 1.15	2.23 ± 1.11	2.17 ± 0.42	*F* = 0.078 *p* = 0.780	*F* = 0.319 *p* = 0.573	*F* = 0.037 *p* = 0.847
	Maximum power (W)	14.28 ± 3.83	11.97 ± 6.50	14.70 ± 8.15	13.62 ± 4.59	*F* = 0.740 *p* = 0.392	*F* = 1.973 *p* = 0.163	***F*** **=** **0.614** ***p*** **=** **0.003**
	Positive Work (J kg^−1^)	0.30 ± 0.86^a,c^	0.34 ± 0.23^b,c^	0.32 ± 0.16^a^	0.23 ± 0.96^b^	*F* = 2.510 *p* = 0.116	*F* = 0.551 *p* = 0.460	***F*** **=** **4.453** ***p*** **=** **0.037**
	Negative Work (J kg^−1^)	0.47 ± 0.10^a,c^	0.45 ± 022^b,c^	0.42 ± 0.21^a^	0.50 ± 0.20^b^	*F* = 0.014 *p* = 0.905	*F* = 0.548 *p* = 0.461	*F* = 1.666 *p* = 0.200
Hip	ROM (°)	43.42 ± 6.60^a^	40.63 ± 4.08	35.74 ± 2.75^a^	37.64 ± 7.98	***F*** **=** **21.311** ***p*** **<** **0.010**	*F* = 0.146 *p* = 0.703	***F*** **=** **4.105** ***p*** **=** **0.046**
	Peak angular velocity (rad s^−1^)	0.71 ± 0.32^a,c^	1.68 ± 1.33^b,c^	2.13 ± 1.68^a^	2.04 ± 1.19^b^	***F*** **=** **12.939** ***p*** **<** **0.001**	*F* = 3.193 *p* = 0.770	***F*** **=** **4.550** ***p*** **=** **0.035**
	Peak Moment (N m kg^−1^)	3.79 ± 0.68^c^	3.25 ± 0.69^c^	3.67 ± 0.86	3.58 ± 0.55	*F* = 0.565 *p* = 0.454	***F*** **=** **4.905** ***p*** **=** **0.029**	*F* = 2.644 *p* = 0.107
	Maximum power (W)	10.85 ± 2.08	10.06 ± 4.79	11.90 ± 4.15	10.72 ± 2.62	*F* = 1.423 *p* = 0.236	*F* = 1.914 *p* = 1.170	*F* = 1.002 *p* = 0.780
	Positive Work (J kg^−1^)	0.82 ± 1.99	0.77 ± 0.36	0.97 ± 0.33	0.85 ± 0.26	*F* = 3.619 *p* = 0.060	*F* = 1.944 *p* = 0.166	*F* = 0.334 *p* = 0.565
	Negative Work (J kg^−1^)	0.14 ± 0.08	0.14 ± 0.15	0.13 ± 0.10	0.14 ± 0.10	*F* = 0.044 *p* = 0.835	*F* = 0.024 *p* = 0.876	*F* = 0.072 *p* = 0.789

Statistical significance was set to *p* < 0.05. The significant differences in interaction effect were based on the results of Bonferroni *post hoc* tests (*α* = 0.008). The bold represented significant differences. A means bionic shoes and B means neutral shoes. L1 represents experienced runners, L2 represents novice runners. S means main effect of shoes, L means main effect of running experience, and S × L means interaction effect.

^a^ Represents significant differences between A and B in group L1.

^b^ Represents significant differences between A and B in group L2.

^c^ Represents significant difference between L1 and L2 in A.

^d^ Represents significant difference between L1 and L2 in B.

### 3.2 Effects of exercise level

Regarding the exercise level, significant differences also can be seen at the ankle, knee and hip joints, as shown in Table 1. The specific differences are as follows: (1) In experienced runners, the peak angular velocity of ankle joint (*F* = 8.222; *p* < 0.010) and knee joint (*F* = 21.918; *p* < 0.010) were bigger than novice runners. (2) Both in bionic shoes and neutral shoes, the experienced runners showed greater peak moment in ankle joint (*F* = 13.044; *p* < 0.010) and hip joint (*F* = 4.905; *p* < 0.050). (3) Besides, experienced runners have significantly greater maximum power (*F* = 16.465; *p* < 0.001) of ankle joint than novice runners, and the range of moment in novice runners were bigger than that in experienced runners. The waveform of moment and joint power of ankle, knee and hip joints are shown in Figure 3.

### 3.3 Interaction effects

The interaction between the shoe condition and running experience induced a significant effect on the knee and hip joint (Table 1). For knee joint, when wearing bionic shoes, the maximum knee power of experienced runners was significantly higher than that of novice runners (*F* = 0.614, *p* < 0.050), and the positive knee power of bionic shoes was significantly higher than that of neutral shoes in novice runners (*F* = 4.453, *P* < 0.050). For hip joint, the ROM of bionic shoes of experienced runners was significantly greater than that of neutral shoes (*F* = 4.105, *p* < 0.050), while wearing bionic shoes, the maximum hip angular velocity of novice runners was significantly greater than that of experienced runners (*F* = 4.550, *P* < 0.050).

### 3.4 SPM1D effects

The results of SPM1D are as shown in Figure 3, significant differences were seen in ankle, knee and hip joints. Regarding the ankle joint, when wearing bionic shoes, the motion angle of the ankle plantarflexion/dorsiflexion decreased by 79%−99% (*p* < 0.050), the ankle angle velocity increased by 30%−44% (*p* < 0.010) and 52%−99% (*p* = 0.010), the ankle moment increased by 25%−57% (*p* < 0.001), and the peak power of ankle increased by 22%−46% (*p* < 0.010), 53%−83% (*p* < 0.010) and 89%−99% (*p* < 0.010). While the novice runners' motion angle of the ankle plantarflexion/dorsiflexion decreased by 18%−91% (*p* < 0.010), the ankle angle velocity increased by 30%−44% (*p* < 0.01) and 52%−99% (*p* < 0.010), the ankle moment decreased by 1%−34% (*p* < 0.010), and the ankle positive power de-creased by 1%−30% (*p* < 0.050).

Regarding the knee joint, when wearing bionic shoes, the motion angle of the knee flexion/extension decreased by 29%−34% (*p* < 0.050) and 61%−99% (*p* < 0.050), the knee angle velocity increased by 30%−81% (*p* < 0.010), the knee moment decreased by 64%−94% (*p* < 0.010), the positive power increased by 38%−62% (*p* < 0.010) and 79%−93% (*p* < 0.010), while the novice runners' angle velocity of knee flexion/extension increased by 19%−34% (*p* < 0.010) and decreased by 76%−93% (*p* < 0.010).

For the hip joint, when wearing bionic shoes, the motion of angle of hip flexion/extension decreased by 66%−99% (*p* < 0.010), the hip joint angular velocity increased by 38%−54% (*p* < 0.050) and 83%−96% (*p* < 0.010), the hip joint moment decreased by 60%−99% (*p* < 0.010), the hip joint peak power increased by 30%−42% (*p* < 0.010) and de-creased by 66%−99% (*p* < 0.050). And the hip joint angle increased by 0%−46% (*p* < 0.010) and decreased by 91%−99% (*p* < 0.010) for novice runners.

## 4 Discussion

The purpose of this study was to investigate the difference of lower limb biomechanics between experienced runners and novice runners to compare the effect of wearing bionic shoes with normal shoes, respectively. Although few studies have explored the biomechanics of wearing bionic shoes, our scientific study will provide a novel perspective to this field, offering fresh insight for future investigation. The results of this study show that bionic shoes have little impact on running performance, and the unstable conditions of bionic shoes have a good training effect on the relevant muscles of the body may have the potential to better maintain body stability. Compared to novice runners, experienced runners perform greater moments and power in the lower limb joints, with primary differences being observed in ankle and hip joint. With better running experience, runners can change their body status in time during different sports to adapt to the changing external environment to the greatest extent, and also have better sports performance (22).

### 4.1 Shoes

In terms of the effects of footwear, it is clear that hip flexion and ankle plantar/dorsiflexion have larger ROM values when wearing bionic shoes, as shown in Figure 3, while neutral shoes have smaller ROM values. Research has point out that the change of ankle torque and ROM have obvious effect on the work efficiency of the power output, energy consumption, and if the joint torque is too large during the movement, it is easy to lead to muscle strain, ligament damage and other problems (51). In this experiment, the relevant indexes also changed when wearing different shoes, so whether different shoes have an effect on muscle injury and output power is worth exploring. Some scholars have pointed out that the design of running shoes may affect the movement control system of the human body during running (52), and runners will adapt to different running shoe conditions by changing the activities of the body muscles. Previous studies have shown that unstable shoes can lead to changes in kinematic data of lower limb joints (53, 54). McCarthy. C et al. (55) found a significant increase in ankle range of motion during the support phase of running compared to traditional running shoes. We found the same result in our study. Compared with traditional running shoes, the sole thickness of bionic shoes is small, the difference between the front sole and the back sole is close to zero, and the contact area of the foot is increased when the foot lands in the middle, which may also increase the participation of the ankle joint in absorbing energy when hitting the ground. At the same time, from a training point of view, we also want to add ROM to get better training results. The ankle torque of bionic shoes is also significantly greater than that of neutral shoes, which is consistent with previous research results (56). Due to the thin soles of bionic shoes, the support provided by runners during running is insufficient, and greater force must be generated to meet the needs of ankle joint stability during exercise. Therefore, when running in bionic shoes, the probability of injury will also increase. Other studies have shown that injured runners have significantly less hip ROM than uninjured runners (57). Generally, for runners, larger hip and ankle ROM is the body's protective way to avoid injury. Less hip motion usually the hip is too tight, affecting the lower limb blood circulation, easy to produce hip pain, knee and lumbar spine will also produce compensation, resulting in unnecessary sports injuries. In this study, the peak overall angular velocity of the hip joint in bionic shoes is low, with the hip joint serving as a crucial center for maintaining human stability. The study found that there is a significant correlation between the activities of various muscle groups of the hip joint and the injuries caused by overuse of the lower limbs (58). To sum up, and combine with our experimental results (greater hip range of motion, smaller peak angular velocity, positive, and negative work values), we believe that bionic shoes provide more protection for the hip.

### 4.2 Experience

As an athletic skill that refines with experience, similar to how fatigue influences performance, running experience can also impact lower limb coordination (59). In terms of ankles, whether bionic shoes or neutral shoes, we found that experienced runners had significantly higher ankle angular speed than novices, and novices had slightly lower plantar-flexion/dorsiflexion angles, ROM and plantar-flexion moments than experienced runners. Therefore, for novice runners, good ankle muscle strength is very important, the increase of muscle strength around the ankle joint can not only improve the body's ability to withstand load by increasing the ultimate strength of the muscle, but also reduce the internal load of the joint and muscle overuse. In addition, we suggest that novice runners should gradually increase the amount of exercise training during the exercise process to avoid violent fluctuations in training to reduce injuries. Previous studies have shown that the dorsiflexion angle decreases during the support period after running for more than 5 km, which is considered to be the result of fatigue of the dorsiflexor muscle of the foot (60). Therefore, we think that experienced runners can delay the occurrence of fatigue at the same speed and thus perform better. Our results showed that the knee flexion angle of novice runners was smaller than that of experienced runners, and Dierks et al. found that the increase of knee flexion Angle could reduce the occurrence of knee injury (61). Research shows that novice runners are more likely to suffer knee and calf injuries than experienced runners. One possible explanation is that novice runners possess inefficient running mechanisms, resulting in increased strain on musculoskeletal tissues, especially around the knees and tibia (62). In this study, experienced runners experienced a significant increase in the maximum flexion and extension moment of their hip joints. This may be due to more experienced and faster runners having more able with managing their bodies during long-term running (63). Some scholars have pointed out that in long-distance running, experienced runners show greater coordination variability, while novice runners show greater joint and segmental variability (59, 64), so novice runners are more likely to suffer injuries during repetitive running movements. In addition, we found that the positive work of the knee joint of novice runners was significantly greater than that of neutral shoes when wearing bionic shoes, possibly because novice runners lack the ability of controlling muscles. When on unstable occasion (wearing bionic shoes), they need greater work of the knee joint to offset the impact of the ground during exercise. While, different types of running shoes and changes in running conditions will affect the biomechanical characteristics of runners. For example, compared with normal running shoes, cushioned running shoes usually have a thicker sole to play a cushioned effect, so the pressure impact force is significantly different; Different weather and the type of ground on which to run can also have an impact on a runner's performance.

### 4.3 Limitations

There are some limitations to our study. First, our study focused on the support phase during running, because the running support phase is most closely associated with running-related injuries, but the swing phase is also an important part of the running process. It has been shown that the increase of external load such as running speed may change the power contribution from the distal to the proximal end, and the change of kinematics may also affect the lower limb Angle of the sagittal and non-sagittal planes during the swing period (65). Whether the change of footwear will affect the lower limb biomechanical characteristics of runners during the swing period still needs to be further discussed. Secondly, the subjects ran through the force table at a fixed speed for motion capture analysis, lack of comparative analysis at different speeds yet. Different lower limb biomechanical characteristics may emerge at varying speeds whether faster or slower. Third, the subjects who were included in our study were all adult males, and lack of female in our study, thereby, the results obtained were not applicable to female runners. Meanwhile, the sample size in the manuscript is slightly small, and subsequent studies should appropriately increase the sample size to increase the effect size. The results of a single running experiment are insufficient to comprehensively evaluate the advantages of these shoes. Future studies will involve long-term follow-up experiments and the addition of other feasibility indicators, such as injury surveillance, and the actual benefits of the footwear will be validated through thorough data verification over time.

## 5 Conclusion

Compared with neutral shoes, bionic shoes with thinner midsoles will have an impact on joint kinematics and dynamics during the running support period. It can be seen that the motion of the ankle joint and hip joint of the bionic shoes is significantly greater than that of the neutral shoes, and the angular velocity of the hip joint is significantly reduced, which seems to provide good protection for the hip joint. At the same time, experienced runners produced significantly greater joint torque and work than novices, but novices showed a greater indicator difference between the two types of shoes, indicating that novices were more prone to injury during unstable training. Therefore, runners should choose suitable running shoes according to their actual situation and exercise level to achieve the best training effect. The results of this study can help us better understand the knowledge of footwear and running biomechanics, and provide valuable information for injury prevention and athletic performance improvement.

## Data Availability

The raw data supporting the conclusions of this article will be made available by the authors, without undue reservation.
